# Refractory Lichen Amyloidosis Coexisting With Atopic Dermatitis Responsive to Sequential Janus Kinase Inhibitor Therapy: Upadacitinib Followed by Abrocitinib

**DOI:** 10.1002/kjm2.70146

**Published:** 2025-11-29

**Authors:** Yu‐Ting Tsai, Jui Lan, Shang‐Hung Lin

**Affiliations:** ^1^ Department of Dermatology Kaohsiung Chang Gung Memorial Hospital and Chang Gung University College of Medicine Kaohsiung Taiwan; ^2^ Department of Pathology Kaohsiung Municipal Ta‐Tung Hospital Kaohsiung Taiwan

Dear Editor,

1

Lichen amyloidosis (LA), the most common form of primary cutaneous amyloidosis, manifests as intensely pruritic, hyperkeratotic papules, typically on the shins and extensor surfaces. Atopic dermatitis (AD) is a chronic, relapsing inflammatory dermatosis characterized by eczematous lesions and severe pruritus. Coexistence of LA and AD is increasingly recognized and likely reflects shared mechanisms involving immune dysregulation, chronic scratching, and interleukin (IL)‐31–mediated Janus kinase (JAK)‐STAT signaling [1].

A 44‐year‐old woman with a 20‐year history of AD presented with severe generalized pruritus. There was no family history of LA. Physical examination revealed numerous monomorphic, skin‐colored to hyperpigmented papules with a rippled appearance on the extremities (Figure 1A). A biopsy from the left forearm demonstrated hyperkeratosis, hypergranulosis, and irregular psoriasiform hyperplasia of the epidermis, with a positive pseudopalmar sign. The superficial dermis exhibited mild perivascular lymphocytic infiltrates with scattered eosinophils. Expanded dermal papillae contained amorphous pale eosinophilic deposits and melanophages (Figure 1D,E). These findings confirmed LA with chronic eczematous dermatitis. Laboratory tests showed an absolute eosinophil count of 510/μL and an immunoglobulin E level of 1330 U/L, supporting a diagnosis of LA associated with severe AD.

**FIGURE 1:**
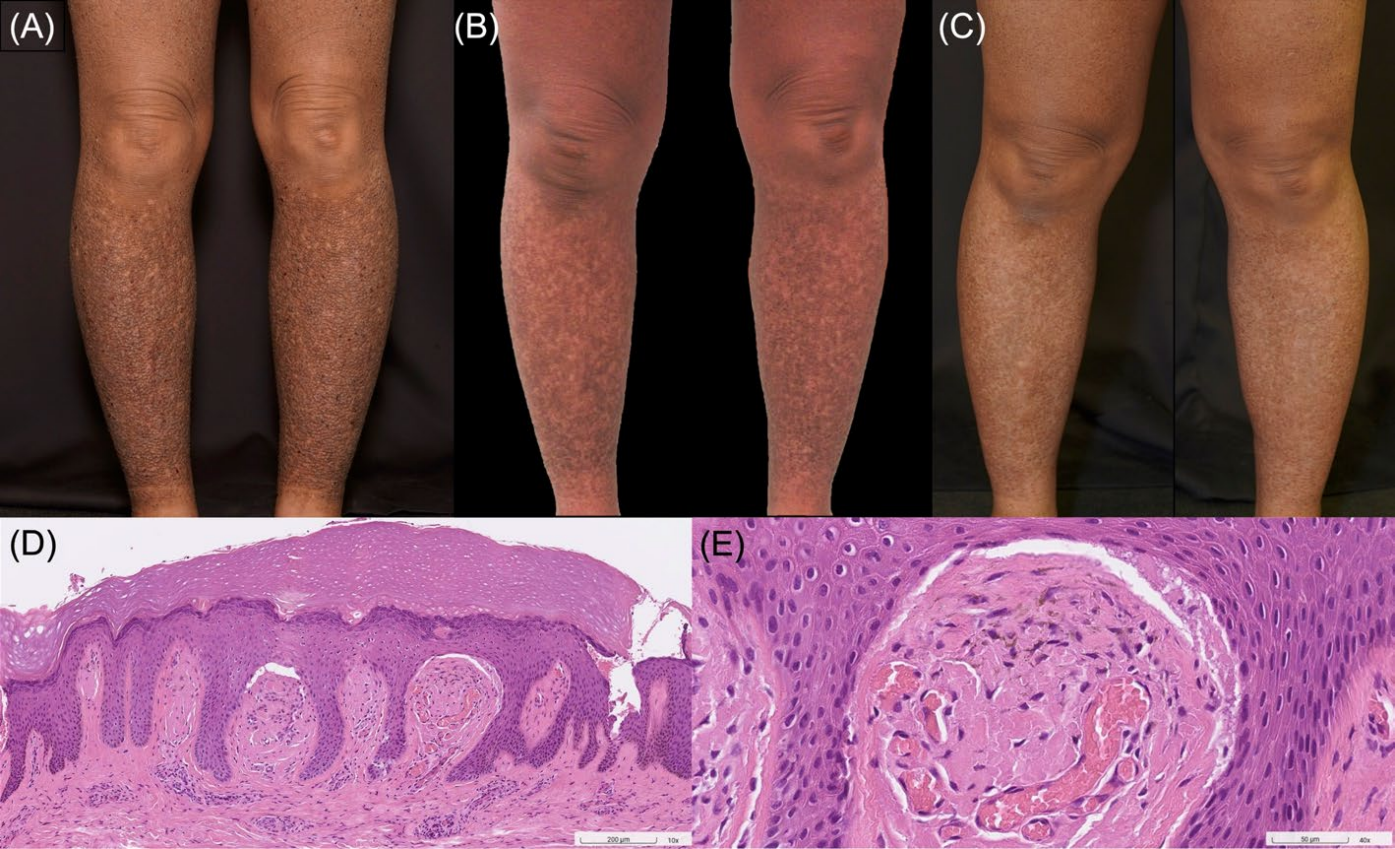
A 44‐year‐old woman presented with numerous monomorphic, skin‐colored to hyperpigmented papules with a rippled appearance on the lower extremities (A). After 1 year of treatment with upadacitinib 15 mg daily, partial improvement was observed (B). Following an additional year of treatment with abrocitinib 200 mg daily, further improvement was noted (C). Hematoxylin and eosin–stained sections showed hyperkeratosis, hypergranulosis, and irregular psoriasiform hyperplasia of the epidermis with a positive pseudopalmar sign, accompanied by mild perivascular lymphocytic infiltrates and scattered eosinophils in the superficial dermis, and amorphous pale eosinophilic deposits with melanophages in the expanded dermal papillae (D and E) (D: ×100, E: ×400).

The patient had previously failed multiple therapies, including topical corticosteroids, methotrexate (5–15 mg/week), cyclosporine (100–300 mg/day), and narrowband UVB phototherapy. Her baseline Eczema Area and Severity Index (EASI) was 22.5, with an Investigator Global Assessment (IGA) score of 3. Initiation of oral upadacitinib (15 mg daily) provided partial relief, reducing EASI to 11.2 and IGA to 1 after 1 year (Figure 1B). Due to persistent pruritus, treatment was switched to oral abrocitinib (200 mg daily), resulting in further improvement: EASI decreased to 5.0, and IGA remained 1 after an additional year (Figure 1C). Although occasional recurrent orolabial herpes simplex virus (HSV) infections occurred after initiating JAK inhibitors, the patient considered that the benefits of these treatments for controlling her disease outweighed this complication. The episodes were mild and successfully managed with intermittent oral acyclovir as needed. Overall, she did not experience other systemic complications during treatment.

The pathogenesis of LA is believed to involve chronic scratching, which induces keratinocyte apoptosis and dermal amyloid deposition. Reported treatments for LA include topical corticosteroids, calcipotriene, phototherapy (narrowband UVB and PUVA), systemic immunosuppressants, retinoids, and biologics such as dupilumab, with variable and often suboptimal outcomes. Central to management is interrupting the itch–scratch cycle, which drives epidermal injury and amyloidogenesis. JAK inhibitors, by targeting pruritogenic and inflammatory cytokines, particularly IL‐4, IL‐13, and IL‐31, significantly alleviate pruritus in AD. Case reports have documented LA improvement with individual JAK inhibitors, including baricitinib 4 mg daily [2], abrocitinib 100 mg daily [1], and upadacitinib at both 15 mg [3] and 30 mg daily [4]. A case of primary cutaneous amyloidosis also improved with tofacitinib 5 mg twice daily [5].

While intra‐class switching between JAK inhibitors has been explored in AD, this appears to be the first reported case of LA with concurrent AD demonstrating a successful sequential response to two JAK1‐selective inhibitors, with improvement after transition from upadacitinib to abrocitinib. Differences in dosage (abrocitinib 200 mg vs. upadacitinib 15 mg), pharmacokinetics, or pharmacodynamics may explain the enhanced response. JAK inhibitors, such as abrocitinib, are generally well tolerated but can be associated with potential adverse events. Serious infections, including HSV, herpes zoster, tuberculosis, and pneumonia, may occur. Cardiovascular and thrombotic complications, such as acute myocardial infarction, cerebrovascular accident, deep vein thrombosis, and pulmonary embolism, have also been reported. Rare cases of malignancies, including lymphoma and nonmelanoma skin cancers, have been observed.

In conclusion, this case underscores the potential utility of JAK inhibitor switching in refractory dermatoses and highlights the need for further research into individualized, mechanism‐based approaches for LA and other chronic pruritic conditions. Careful monitoring and patient counseling regarding the risks associated with JAK inhibitors are recommended.

## Conflicts of Interest

The authors declare no conflicts of interest.

## Data Availability

The data that support the findings of this study are available from the corresponding author upon reasonable request.
